# 120 Venous Thromboembolism Prophylaxis in Burn Patients

**DOI:** 10.1093/jbcr/irae036.119

**Published:** 2024-04-17

**Authors:** Eloise Stanton, Haig A Yenikomshian, Justin Gillenwater

**Affiliations:** Keck School of Medicine of USC, Los Angeles, CA; University of Southern California, Los Angeles, CA; Keck Medicine of USC, Los Angeles, CA; Keck School of Medicine of USC, Los Angeles, CA; University of Southern California, Los Angeles, CA; Keck Medicine of USC, Los Angeles, CA; Keck School of Medicine of USC, Los Angeles, CA; University of Southern California, Los Angeles, CA; Keck Medicine of USC, Los Angeles, CA

## Abstract

**Introduction:**

Venous thromboembolism (VTE) is extensively studied in trauma patients, but burn patients require special consideration due to their unique pathophysiology and management strategies. Currently, there are no specific prophylaxis recommendations to help mitigate VTE in burn patients.

**Methods:**

The US National Trauma Data Bank (NTDB) was analyzed to identify patients with burn encounters from 2017-2021 using ICD-10 event codes. Our primary outcome measure was incidence of VTE, which included deep venous thrombosis and pulmonary embolism. Secondary outcome measures included need for blood transfusion and need to return to the operating room due to hemorrhage from skin/soft tissues/extremities. Multivariable regressions were conducted to evaluate the relationship between various VTE prophylaxis types and timing of VTE prophylaxis and VTE incidence while controlling for age, sex, %TBSA and inhalation injury.

**Results:**

A total of 326,614 patients with burn injuries were identified in the NTDB during the study period, of whom 5,604 (1.7%) had a VTE event during their hospital admission. The majority of patients did not receive VTE prophylaxis (54.1%). Of those that did, the most commonly used VTE prophylaxis was LMWH (37.9%), followed by unfractionated heparin (4.1%). When controlling for age, sex, inhalation injury, and %TBSA, unfractionated heparin and LMWH were significantly associated with an increased risk of VTE (heparin: OR=1.8 95% CI 1.3-2.5, p < .001; LMWH: OR=1.5 95% CI 1.2-1.8, p < .001). Longer time to VTE prophylaxis was significantly associated with increased odds of VTE when controlling for the same covariates (OR=1.04 95% CI 1.03=1.07, p < .001). Neither VTE prophylaxis type nor timing of VTE prophylaxis administration was significantly associated with an increased need for blood transfusion. Heparin was the only VTE prophylaxis type significantly associated with increased odds of returning to the OR for a bleeding-related complication (p < .001).

**Conclusions:**

A majority of burn patients in the NTDB did not receive VTE chemoprophylaxis, and counterintuitively, patients who received VTE chemoprophylaxis had higher rates of VTE than patients who did not, despite controlling for known VTE risk factors. In patients who did get chemoprophylaxis, unfractionated heparin is associated with a higher rate of VTE and surgical bleeding complications when compared with LMWH. Delayed initiation of VTE prophylaxis correlates with a higher likelihood of VTE occurrence. These findings underscore the importance of better studying and developing specialized VTE prevention guidelines for burn patients to enhance their care and outcomes.

**Applicability of Research to Practice:**

Prompt initiation of VTE prophylaxis is essential to reduce the risk of VTE among burn patients, highlighting the importance of timely interventions. Developing specialized VTE prevention guidelines for this population can enhance their care and outcomes.

